# Integrated omics analysis identifies GSTA1 and ALDH1A1 as candidate biomarkers for low-level albuminuric diabetic kidney disease: a mixed prospective-retrospective cross-sectional study

**DOI:** 10.3389/fendo.2026.1978947

**Published:** 2026-10-19

**Authors:** Xiaohong Chen, Wei Su, Tao Yang, Jianghua Huang, Bitao Wu, Qiang Yang, Ying Jin, Jie Tang, Pei Yang, Yuwei Yang

**Affiliations:** 1 Mianyang Central Hospital, School of Medicine, University of Electronic Science and Technology of China, Mianyang, China; 2 NHC Key Laboratory of Nuclear Technology Medical Transformation (Mianyang Central Hospital), Mianyang, China; 3 Santai County People’s Hospital, Mianyang, China

**Keywords:** candidate biomarker, diabetic kidney disease, integrated analysis, low albuminuria, metabolomics, proteomics

## Abstract

**Objective:**

Diagnosing a non-albuminuric diabetic kidney disease (DKD) remains challenging, primarily attributable to the absence of specific biomarkers. This study identifies potential biomarkers via integrated proteomic-metabolomic analysis, to provide evidence for optimizing DKD management.

**Methods:**

This combined retrospective and prospective observational study were conducted at the Department of Endocrinology. All patients with diabetes mellitus (DM) between 2023 and 2024 were retrospectively reviewed and classified into normo-, low-, high-, and very-high albuminuric DKD (subdivided NADKD, LADKD, HADKD, and VADKD) and simple DM for prevalence analysis. Newly diagnosed subdivided NADKD, LADKD, HADKD, and VADKD patients from January to March 2025 were prospectively enrolled to explore differential proteins via integrated proteomic-metabolomic analysis. Finally, 270 simple DM, 270 LADKD and 270 HADKD/VADKD patients were randomly enrolled to preliminarily verify the diagnostic performance of candidate protein.

**Results:**

The retrospective investigation revealed that non-albuminuric DKD patients are predominantly of advanced age, and the subdivided NADKD is rare, accounting for 0.7% of all DM patients and 1.6% of all DKD patients. In the prospective omics cohort, LADKD differed metabolically from simple DM and albuminuric DKD across 33 metabolic pathways involving 29 differential proteins. These proteins were primarily involved in extracellular exosome, cytosol, nucleus, membrane, extracellular space, and cytoplasm. In the KEGG enrichment network, they primarily participate in key pathways including Metabolic pathways, Biosynthesis of amino acids, Ribosome, One carbon pool by folate, Coronavirus disease, and Carbon metabolism. After screening, seven candidate proteins obtained an excellent classification performance and achieved a strong 5-fold cross-validation mean AUC exceeding 0.8 in the XGBoost classifier. Finally, the SHAP analysis confirmed that GSTA1 and ALDH1A1 ranked higher in feature importance across on both the training set (SHAP ± SE: 0.219 ± 0.033, 0.203 ± 0.037) and testing set (SHAP ± SE: 0.229 ± 0.050, 0.174 ± 0.038).

**Conclusion:**

This study proposes a potential disease trajectory of DM→LADKD→ albuminuric DKD, which appears to contrast with the older age profile observed in non-albuminuric DKD patients. Glutathione S-transferase A1 and aldehyde dehydrogenase 1 alpha 1 emerge as the most biologically significant candidate biomarkers, collectively implicating tubular injury as a potential trajectory in LADKD.

## Introduction

Diabetic kidney disease (DKD), the most common microvascular complication of diabetes mellitus (DM) (1, 2) and a leading cause of end-stage renal disease (3, 4), is defined by the American Diabetes Association (ADA) as chronic kidney disease caused by diabetes, primarily characterized by a urinary albumin-to-creatinine ratio (UACR)≥30 mg/g and/or an estimated glomerular filtration rate (eGFR)<60 mL/min/1.73m^2^, with these abnormalities persisting for more than 3 months (5). Albuminuria (UACR≥30 mg/g) is widely recognized as the preferred DKD screening criterion by most researchers. However, the 1982 discovery of normoalbuminuric DKD (NADKD) in patients with albumin excretion rate between 30 and 300 mg/d (Equivalent to UACR 3-30mg/g) (5) has called into question the validity of relying solely on albuminuria for DKD screening. In the past 30 years, NADKD has been reported to account for 20%-70% of DKD patients across different regions (6–8). A recent meta-analysis shows nearly half of patients with diabetes-related renal impairment have DKD without albuminuria (9). Therefore, in the current diagnostic framework that prioritizes albuminuria screening, NADKD is inevitably underdiagnosed.

Meanwhile, diagnosing NADKD is challenging and requires caution. It demands ruling out chronic kidney disease from non-diabetic factors, and available diagnostic markers are extremely limited, relying mainly on reduced glomerular filtration rate (GFR). Measured GFR (mGFR) is not routinely available, and guideline-recommended eGFR methods deviate significantly from actual GFR in many cases (10–12), often leading to misdiagnosis and missed diagnosis. Thus, identifying effective NADKD biomarkers is an urgent priority. Given the protein structural modifications, altered protein levels (13, 14) and metabolic disturbances (15–17) observed in DKD patients, proteomics and metabolomics represent promising approaches for biomarker discovery. While most previous DKD proteomic studies have successfully differentiated albuminuric DKD from simple DM (18–21), proteomic research specifically targeting NADKD remains scarce. Nevertheless, several proteins have been reported to assist NADKD diagnosis, including zinc-α2-glycoprotein (22), liver-type fatty acid-binding protein (23), and heart-type fatty acid-binding protein (24). The association between neutrophil gelatinase-associated lipocalin (NGAL) and NADKD has also been confirmed: NGAL typically elevates before clinical albuminuria develops in DM patients (25), and its levels rise progressively as UACR increases from 10 mg/g to 30 mg/g (26). By comparison, several domestic and international metabolomics studies on NADKD have been conducted, all indicating significant differences in urinary metabolic disturbances between albuminuric DKD and NADKD (27, 28). However, the clinical application of these proteins and metabolites still needs further investigation.

Additionally, the concept of NADKD has faced challenges from the updated albuminuria categories. In 2009, the NKF/FDA Workshop proposed a four-category classification for albuminuria (29), subdividing the original “normal albuminuria” (UACR<30mg/g) into “low albuminuria” (UACR 10-29mg/g) and the strict “normal albuminuria” (UACR<10mg/g). This means the traditional NADKD defined by UACR<30mg/g actually covers the two subgroups: low-albuminuric DKD (UACR 10–29 mg/g) and NADKD (UACR<10mg/g). For clarity, the subgroup with UACR 10–29 mg/g and eGFR<60mL/min/1.73m^2^ is defined as LADKD, and the subgroup with UACR<10 mg/g and eGFR<60mL/min/1.73m^2^ is defined as subdivided NADKD. Previous studies rarely distinguished between subdivided NADKD and LADKD, leaving three core questions to clarify: 1) What are the proportions of subdivided NADKD and LADKD? 2) Are the two conditions unique DKD subtypes or rather different clinical stages of DKD progression? 3) Given methodological influences on UACR and eGFR measurements, does the subdivided NADKD truly exist? The present study was designed to address these gaps.

Therefore, this study was divided into three phases: 1) Retrospective percentage analysis of subdivided NADKD and LADKD patients; 2) Integrated proteomic-metabolomic analysis of mGFR-validated subdivided NADKD and LADKD patients to identify potential biomarkers; 3) Screening candidate markers for application validation.

## Methods

### Study design and patients

This study was conducted at Mianyang Central Hospital, School of Medicine, University of Electronic Science and Technology of China, and it was divided into three sequential phases: prospective proteomic and metabolomic profiling, retrospective cross-sectional survey, and prospective preliminary validation.

During the prospective proteomic and metabolomic profiling phase: following guideline requirements of a minimum five-year diabetes duration, only simple type 2 DM patients with ≥5-year duration and newly diagnosed DKD patients were enrolled during January to March 2025. After a three-month follow-up with monthly UACR and eGFR measurements, participants were preliminary classified into five groups: simple DM, subdivided NADKD, LADKD, HADKD, and VADKD. Since calculated eGFR can deviate up to 25% from actual GFR (10–12) and UACR variability, dynamic ^99m^Tc-DTPA clearance and UACR repeat testing were performed to confirm or correct subdivided NADKD and LADKD cases with UACR between 5 to 15 mg/g and eGFR between 45 to 75 mL/min/1.73m^2^. Final cohort included: 34 simple DM (≥5-year duration), 28 LADKD, 21 HADKD, and 17 VADKD patients. Only one subdivided NADKD case was collected, so it was excluded from omics profiling for insufficient statistical power.

During the retrospective cross-sectional phase: to verify the rarity of subdivided NADKD, a total of 10179 patients managed by the Department of Endocrinology from January 2023 to December 2024 were continuous reviewed. All patients had at least three serial measurements of UACR and eGFR, obtained at approximately monthly or bimonthly intervals. Only the most recent three records were used for the percentage analysis of subdivided NADKD and LADKD patients.

During the preliminary validation phase: 810 patients (270 with simple DM, 270 with LADKD, and 270 with HADKD/VADKD) were randomly employed from April to December 2025 and divided into training and testing sets (n=567 and 243, 189 and 81 per disease group, respectively) at a ratio of 7:3 to evaluate the application performance of differential proteins.

The inclusion criteria were as follows: 1) Type 2 diabetes diagnosis as per the American Diabetes Association’s 2024 guidelines (30); 2) Simple DM group exhibited an eGFR>60ml/min/1.73m^2^ and UACR ≤ 30mg/g in at least 2 of 3 consecutive monthly tests, and had no microvascular complications; 3) Subdivided NADKD group exhibited an eGFR ≤ 60ml/min/1.73m^2^ in at least two of three consecutive monthly tests and UACR<10mg/g in three consecutive monthly tests; 4) LADKD group exhibited a UACR = 10.0-29.9mg/g and eGFR ≤ 60ml/min/1.73m^2^ in at least two of three consecutive monthly tests; 5) HADKD group exhibited a UACR = 30.0-299.9mg/g in at least two of three consecutive monthly tests, irrespective of the eGFR; 6) VADKD group exhibited a UACR≥300mg/g in at least two of three consecutive monthly tests, irrespective of the eGFR; 7) Retinopathy, foot ulcers, or other microvascular complications may coexist with any DKD stage (31); 8) For simple DM, subdivided NADKD and LADKD cases in omics testing cohort, meeting 2 out of 3 tests with UACR 5-15mg/g and eGFR 45–75 mL/min/1.73m^2^, UACR and GFR were confirmed via repeat testing and dynamic ^99^Tc-DTPA clearance after the third follow-up, respectively.

The exclusion criteria were as follows: 1) Diabetic history less than 5 years; 2) Use of nephrotoxic drugs or comorbidities affecting urinary albumin excretion or GFR; 3) Known non-diabetic kidney disease; 4) Pregnant women.

### Measured GFR and UACR repeat testing of patients with subdivided NADAD and LADKD

Dynamic 99mTc-DTPA clearance was performed on a Symbia Intevo 6 SPECT/CT system (Siemens, Germany). Measuring procedure: patient was placed in supine position for posterior acquisition; the attenuation correction unit syringe was counted for 30 seconds before injection; after intravenous access establishment, ^99m^Tc-DTPA was injected in a bolus fashion, followed immediately by dynamic acquisition (30 frames at 2s/frame, then 80 additional frames at 15s intervals); post-imaging, the syringe was counted again for 60 seconds; renal contours and background regions were delineated via region-of-interest counting. Using the Gates formula, patient height, weight, and pre-injection syringe attenuation correction coefficient were input to calculate bilateral GFR (unit: mL/min/1.73m^2^), whose average was taken as mGFR. UACR was automatically calculated and output by the A25 automatic protein analyzer (BioSystem, Spain) based on the detection results of urinary albumin via immunoturbidimetry and urinary creatinine via sarcosine oxidase method. All reagent kits used in the test were provided by Chongqing Biotechnology Co., Ltd. If UACR<10mg/g and mGFR<60mL/min/1.73m^2^ confirmed the subdivided NADKD diagnosis, if UACR 10-29.9mg/g and mGFR<60mL/min/1.73m^2^ confirmed the LADKD diagnosis, if mGFR>60mL/min/1.73m^2^ revised the diagnosis to simple DM (**Figure 1**).

**Figure 1:**
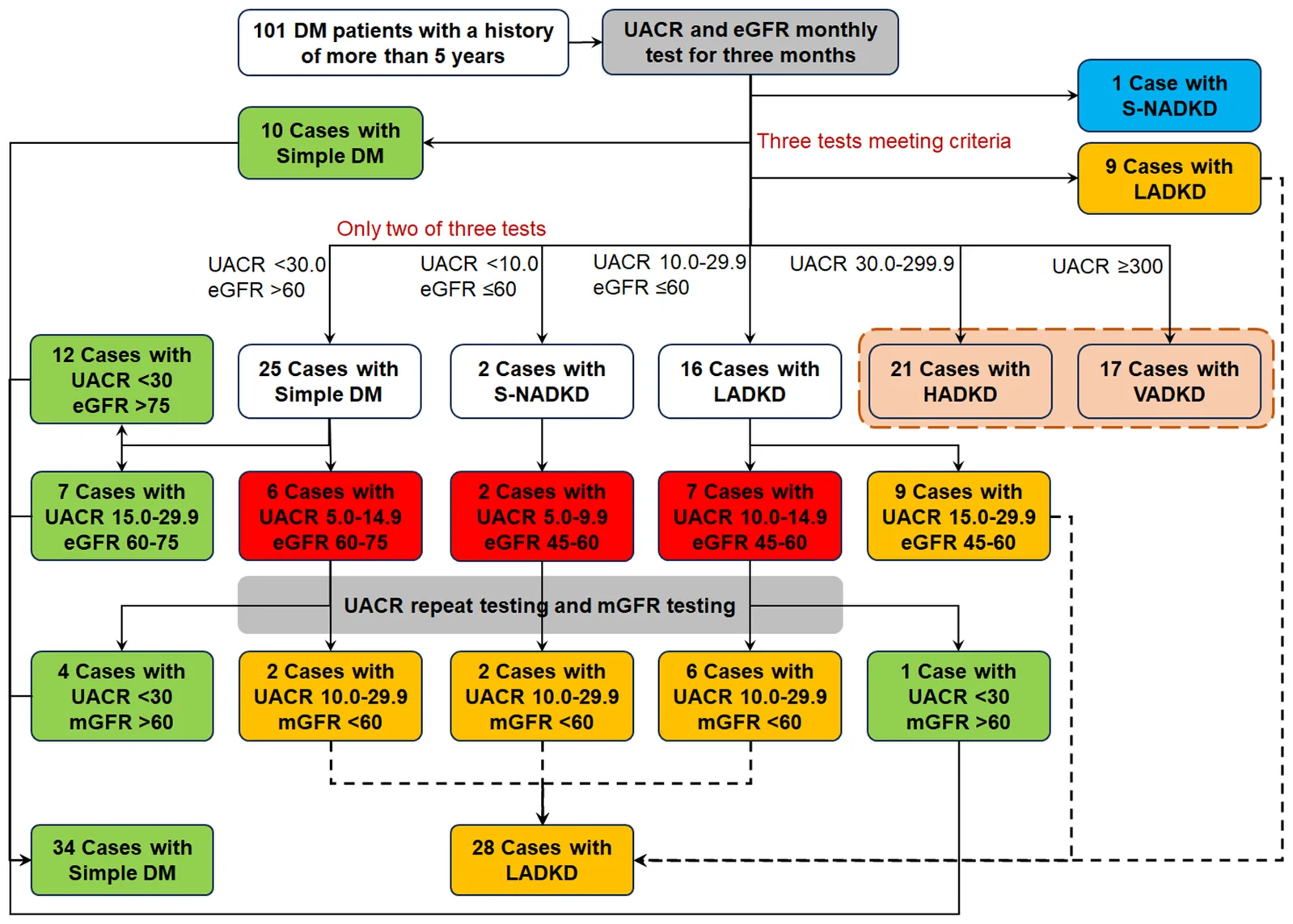
Omics testing patient enrollment and UACR and mGFR confirmation procedures. Note: DM, diabetes mellitus; UACR, urinary albumin-to-creatinine ratio; eGFR, estimated glomerular filtration rate; NADKD, subdivided normoalbuminuric diabetic kidney disease (UACR<10mg/g); LADKD, low-level albuminuric diabetic kidney disease (UACR = 10-29mg/g); HADKD, high-level albuminuric diabetic kidney disease; VADKD, very-high-level albuminuric diabetic kidney disease; mGFR, measured glomerular filtration rate. Based on predefined criteria, 6 cases with simple DM, 2 cases with subdivided NADKD, and 7 cases with LADKD were included for reevaluation. Of these, 2 cases with simple DM and 2 cases with subdivided NADKD were reclassified to LADKD, while 1 case of LADKD was reclassified to simple DM.

### Sample collecting and processing

Aliquots of 3.0mL fasting blood samples were collected from participants in SST-II vacuum tubes (BD, USA) for proteomic, metabolomic, and differential protein measurements. Serums were separated by centrifugation at 1500×g for 10 minutes and stored at -80 °C for up to 6 months. The serum samples collected during the prospective omics profiling phase were shipped to Suzhou BioNovoGene Biomedical Technology Co., Ltd. for proteomic and metabolomic profiling. The serum samples collected during the preliminary validation phase were used for differential protein detections.

### Data-independent acquisition proteomics

Proteomic profiling was conducted on a Vanquish NeoTM Ultra-high performance liquid chromatography/Orbitrap AstralTM Mass Spectrometer system (ThermoFisher Scientific, USA). Liquid chromatography conditions: μPAC High Throughput column (Micropac, Garland, TX, USA) held at 40°C, flow rate 1.8 μL/min. Mobile phase: (A) 0.1% formic acid in water, (B) 0.1% formic acid in 80% acetonitrile. Separation gradient: 0~4 min, 4%~20% B; 4~5.8 min, 20%~35% B; 5.9~6.2 min, 35%~99% B; 6.3~6.9 min, 99% B; 7~8 min, 99%~4% B. Mass spectrometry parameters (positive ion detection mode): precursor scan range 380–980 m/z, MS1 resolution 240,000 at 200 m/z, normalized AGC target 500%, maximum injection time 5 ms. MS2 used data independent acquisition with: 299 scanning windows, 2 m/z isolation window, 25 eV HCD collision energy, normalized AGC target 500%, maximum injection time 3 ms.

Proteome identification parameters were set as below: Database, Uniprot Reviewed (Swiss-Prot, 20,422 entries, updated March 17, 2023); Enzyme, trypsin; Static modification, carbamidomethyl/+57.021 Da (C); Dynamic modifications, protein N-terminal modification, oxidation/+15.995 Da (M). Database-search-identified proteins met the following requirements: maximum parent ion mass deviation of 10ppm, maximum fragment ion mass deviation of 10ppm, peptide false discovery rate (FDR) < 1%. Compounds with coefficient of variation (CV) < 30% were retained.

### Global targeted metabolomics

Metabonomic analysis was performed via a Vanquish™ Ultra-high performance liquid chromatography/Orbitrap Exploris™ 120 Mass Spectrometer system (ThermoFisher Scientific, USA). Liquid chromatography conditions: ACQUITY UPLC ^®^ HSS T3 column (2.1×100mm, 1.8 µm, Waters, Milford, MA, USA) was maintained at 40°C, with flow rate 0.3mL/min and injection volume 2.0 μL. For LC-ESI (+)-MS, mobile phases were (A2) 0.1% formic acid in water (v/v) and (B2) 0.1% formic acid in acetonitrile (v/v), separated via the following gradient: 0~1 min, 8% B2; 1~8 min, 8%~98% B2; 8~10 min, 98% B2; 10~10.1 min, 98%~8% B2; 10.1~12 min, 8% B2. For LC-ESI(-)-MS, mobile phases were (A3) 5mM ammonium formate and (B3) acetonitrile, separated via the same time and gradient profile. Mass spectrometry conditions: Detection was performed with an ESI ion source in Full MS-dd MS2 (data-dependent MS/MS) acquisition mode. Parameters were set as: sheath gas pressure 40 arb; aux gas flow 10 arb; spray voltage 3.50 kV (ESI+) and -2.50 kV (ESI-); capillary temperature 325 °C; MS1 range m/z 100-1000, resolving power 60000 FWHM; 4 data scans per cycle; MS/MS resolving power 15000 FWHM; normalized collision energy 30%; dynamic exclusion time automatic.

Metabolomic raw data were converted to mzXML format via MSConvert (ProteoWizard, v3.0.8789). Peak detection, retention time alignment and feature filtering were done with the CentWave algorithm in R XCMS package v3.18.0, using these parameters: bandwidth=2s, mass accuracy= ± 15ppm, peak width range= 5-30s, m/z window=0.015Da, minimum adjacent peak m/z difference=0.01Da. This workflow generated a matrix of aligned, quantified metabolic features. Putative compound identification was performed by matching experimental MS/MS spectra and accurate mass data against multiple reference spectral databases: Human Metabolome Database (HMDB), MassBank, LipidMaps, mzCloud, and Kyoto Encyclopedia of Genes and Genomes (KEGG).

### Quality control of omics profiling

To correct for intra-batch variability, a pooled quality control (QC) sample was prepared by mixing 10 μL from each pre-processed proteomics supernatant. The QC sample was injected once every ten analytical runs to monitor and normalize instrument performance. Raw data normalization was performed using support vector machine regression correction based on QC samples to eliminate systematic errors.

### Candidate differential protein screening and measurement

Integrated proteomic-metabolomic analysis was employed for the identification of differentially expressed proteins with potential clinical relevance. Following multicollinearity detection and parameter significance analysis, the serum concentrations of the retained candidate proteins were quantified using enzyme-linked immunosorbent assay (ELISA) on an RT-6100 microplate reader (Rayto, Shenzhen, CHN). The assay kits were provided by either Wuhan Abebio Biotech Co., Ltd. or Shanghai Fantai Biotechnology Co., Ltd. Their specific kit catalog numbers were as follows: vascular endothelial growth factor D (VEGFD), FT-P33400R; aldehyde dehydrogenase 1 A1 (ALDH1A1), FT-P32193R; plasminogen (PLG), FT-P32469R; glutathione S-transferase alpha 1 (GSTA1), FT-P31639R; adenosyl homocysteinase (AHCY), FT-P37963R; syndecan-4 (SDC4), FT-P31528R; ribosomal protein L37a (RPL37A), AE21490HU; ras-related C3 botulinum toxin substrate 1 (RAC1), FT-P36332R; beta-1,3-galactosyltransferase 6 (B3GALT6), FT-P36140R; and osteomodulin (OMD), FT-P37652R.

### Statistical analysis

Bioinformatic analysis was conducted on the Wukong Omics Cloud Platform (https://wkomics.omicsolution.com/wkomics/main/): metabolomics data was processed via the Metabolomics Data Processing Tandem module, and proteomics data via the MixProTool module, focusing on the results of variance analysis, volcano plot, principal component analysis (PCA), and partial least squares discriminant analysis (PLS-DA). Integrated proteomic-metabolomic analysis applied multi-omics enrichment module analysis, based on metabolic pathways from 11 databases (including KEGG, PID, Reactome, SMPDB, BioCarta, Wikipathways, NetPath, HumanCyc, INOH, PharmGKB, and EHMN databases), to preliminarily screen differentially expressed proteins for GO and KEGG enrichment analysis. Candidate protein biomarkers were screened in R 4.4.3 via LASSO regression (optimal λ.1SE method) and best subset selection (R^2^, AdjR^2^, BIC, and Cp criteria). Their diagnostic value was evaluated by common machine learning classifiers, and feature importance was assessed via Shapley additive explanations (SHAP). If *P* or *Padj* value <0.05, it was considered statistically significant.

## Results

### Percentages of various DKDs in the retrospective cross-sectional cohort

Among the 10179 patients in this retrospective cross-sectional cohort, the percentages of different DKDs were 0.7% for subdivided NADKD, 6.7% for LADKD, 28.0% for HADKD, and 12.0% for VADKD. Among all 4829 DKD patients, the percentage of subdivided NADKD was approximately 1.6%, indicating an extremely rare incidence (**Table 1**).

**Table 1:** Distribution of patients across various DKDs and their primary clinical data.

Item	Simple DM (n=5350)	Subdivided NADKD (n=75)	LADKD (n=678)	HADKD (n=2849)	VADKD (n=1227)	χ^2^, P
Overall proportion (%)	52.6	0.7	6.7	28.0	12.0	/
DKD proportion (%)	/	1.6	14.0	59.0	25.4	/
Gender (M/F)	2986/2364	46/29	355/323	1589/1160	700/527	8.370, 0.079
Age (years)	52 (46, 58)	68 (59, 77)	69 (62, 76)	54 (47, 62)	55 (49, 63)	1280.078, <0.001
Glu (mmol/L)	7.19 (6.06, 9.18)	7.27 (6.17, 9.19)	7.81 (6.69, 10.19)	8.35 (6.93, 10.98)	8.57 (6.79, 11.69)	186.259, <0.001
UACR (mg/g)	11.77 (7.67, 18.49)	6.86 (5.43, 8.97)	18.27 (14.38, 22.68)	69.58 (43.42, 122.86)	1084.76 (534.62, 2592.11)	7508.013, <0.001
eGFR (mL/min/1.73m^2^)	99.0 (89.0, 107.2)	49.5 (40.4, 55.4)	46.2 (38.8, 52.6)	93.1 (75.7, 104.7)	62.2 (38.9, 91.0)	2864.535, <0.001

The results highlighted an extremely low prevalence of subdivided NADKD (0.7%) and a correspondingly low proportion among DKD patients (1.6%), along with the characteristics of advanced age and low eGFR in patients with subdivided NADKD and LADKD.

Patient demographics and primary clinical data: No gender differences were observed across groups (χ2 = 8.370, P>0.05). Advanced ages and lower eGFR levels were observed in subdivided NADKD and LADKD patients. Specially, subdivided NADKD and LADKD patients were significantly older and had lower eGFR than the other three groups (Age: z=9.046~34.207, all Padj<0.001; eGFR: z=-5.229~-43.380, all Padj<0.001), but did not differ from each other (z=1.316 and 0.315, both Padj=0.999); VADKD patients were older and had lower eGFR than HADKD and simple DM (Age: z=4.250 and 10.171, both Padj<0.001; eGFR: z=-6.322 and -11.294, both Padj<0.001); HADKD patients were older and had lower eGFR than simple DM patients (z=7.497 and -4.677, both Padj<0.001).

### Proteomic profiling

Data-independent acquisition proteomics identified 1597 proteins from the UniProt database, of which 1466 were quantifiable. The mean relative standard deviation (RSD) of protein quantification across the four groups ranged between 30% and 40% (**Figure 2A**), indicating acceptable mass spectrometry data quality. While PCA showed overlapping clustering among different DKD groups, PLS-DA revealed clear separation between most groups, and partial overlap was observed between HADKD and VADKD. This indicates significant differences in protein expression profiles between the simple DM, LADKD, and HADKD/VADKD groups (**Figures 2B, C**). The hierarchical cluster plot showed relatively large Euclidean distances between different DKDs and simple DM, but HADKD and VADKD clustered together preferentially, validating the clustering differences among the simple DM, LADKD, and HADKD/VADKD groups (**Figure 2D**). Therefore, separate volcano plot analysiswere performed between LADKD and simple DM, and between LADKD and HADKD/VADKD (fold change: >2 or <0.5, FDR-adjusted P value: <0.05), identifying 61 and 56 differentially expressed proteins respectively (**Figures 2E, F**). After accounting for 20 overlapping proteins, 97 differential proteins were selected for subsequent integrated proteomic-metabolomic analysis.

**Figure 2:**
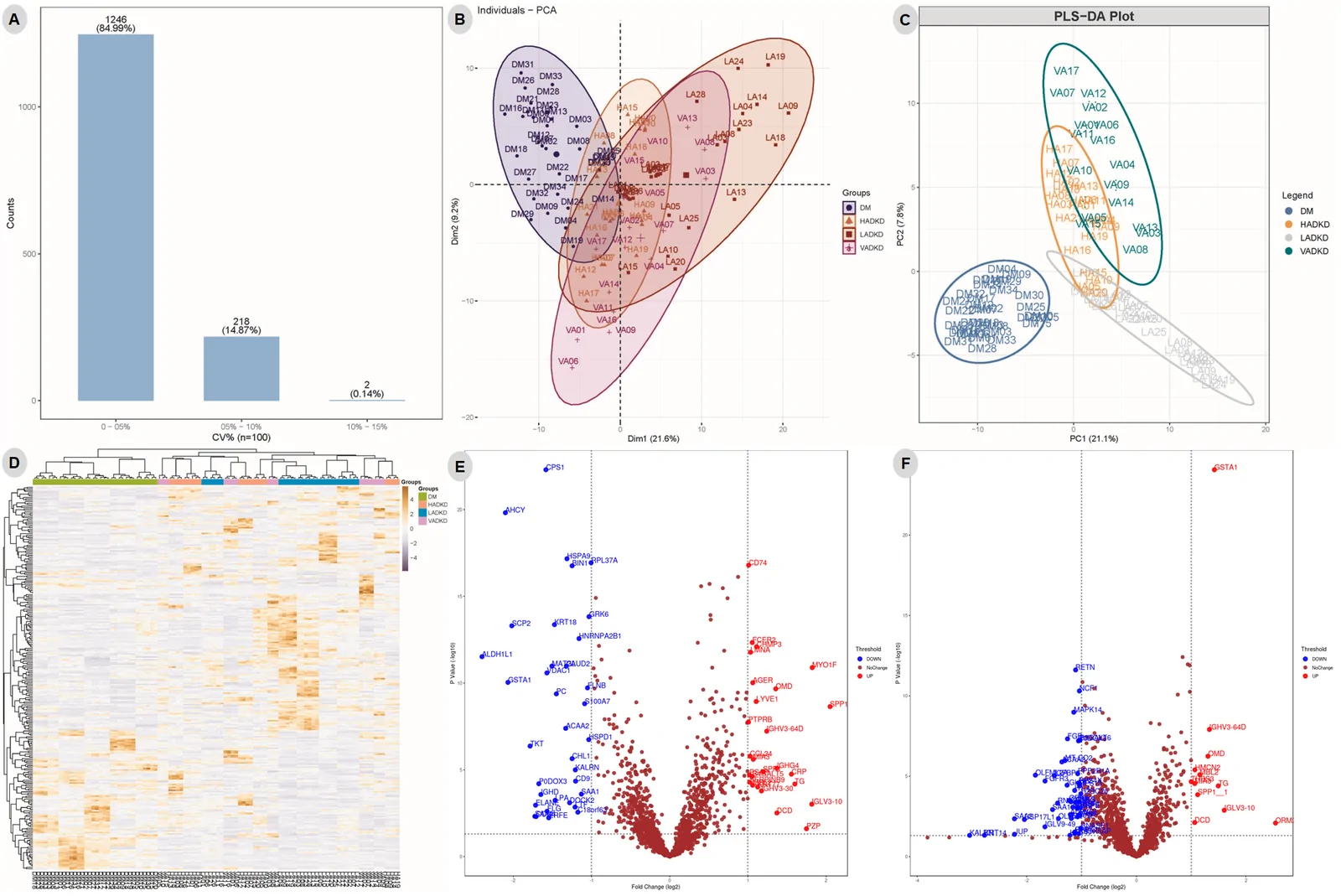
Proteomic profiling focused on the LADKD cohort. **(A)** Coefficient of variation (CV) distribution plot; **(B)** PCA plot; **(C)** PLS-DA plot; **(D)** Hierarchical clustering analysis (HCA) plot; **(E)** Volcano plot between LADKD and simple DM; **(F)** Volcano plot between LADKD and HADKD/VADKD.

### Metabolomic profiling

Global targeted metabolomics revealed that 75.8% and 81.5% of primary metabolites had RSD ≤ 30% in positive and negative ion modes, respectively, confirming good data quality (**Figures 3A, B**). Both PCA and PLS-DA showed clear separation among groups (**Figures 3C–F**), and the hierarchical cluster plot showed distinct groupings (**Figures 3G–J**), revealing significant clustering differences between simple DM, LADKD, HADKD, and VADKD. Ultimately, 1,926 secondary metabolites were successfully identified via database search or standard matching. Among these metabolites, 506 were annotated at Metabolomics Standards Initiative (MSI) Level 1, 1031 were annotated at MSI Level 2, and 389 were annotated at MSI Level 3. For matching proteomics, separate volcano plot analysisselected 84 differential metabolites between LADKD and simple DM, and 83 between LADKD and HADKD/VADKD respectively (fold change: >1.5 or <0.67, FDR-adjusted P value: <0.05). After excluding 5 overlapping differential metabolites, 162 were finalized for subsequent integrated proteomic-metabolomic analysis. For matching proteomics, separate volcano plot analyses identified 84 differential metabolites between LADKD and simple DM, and 83 between LADKD and HADKD/VADKD respectively (fold change: >1.5 or <0.67, FDR-adjusted P value: <0.05) (**Figures 3K, M**).

**Figure 3:**
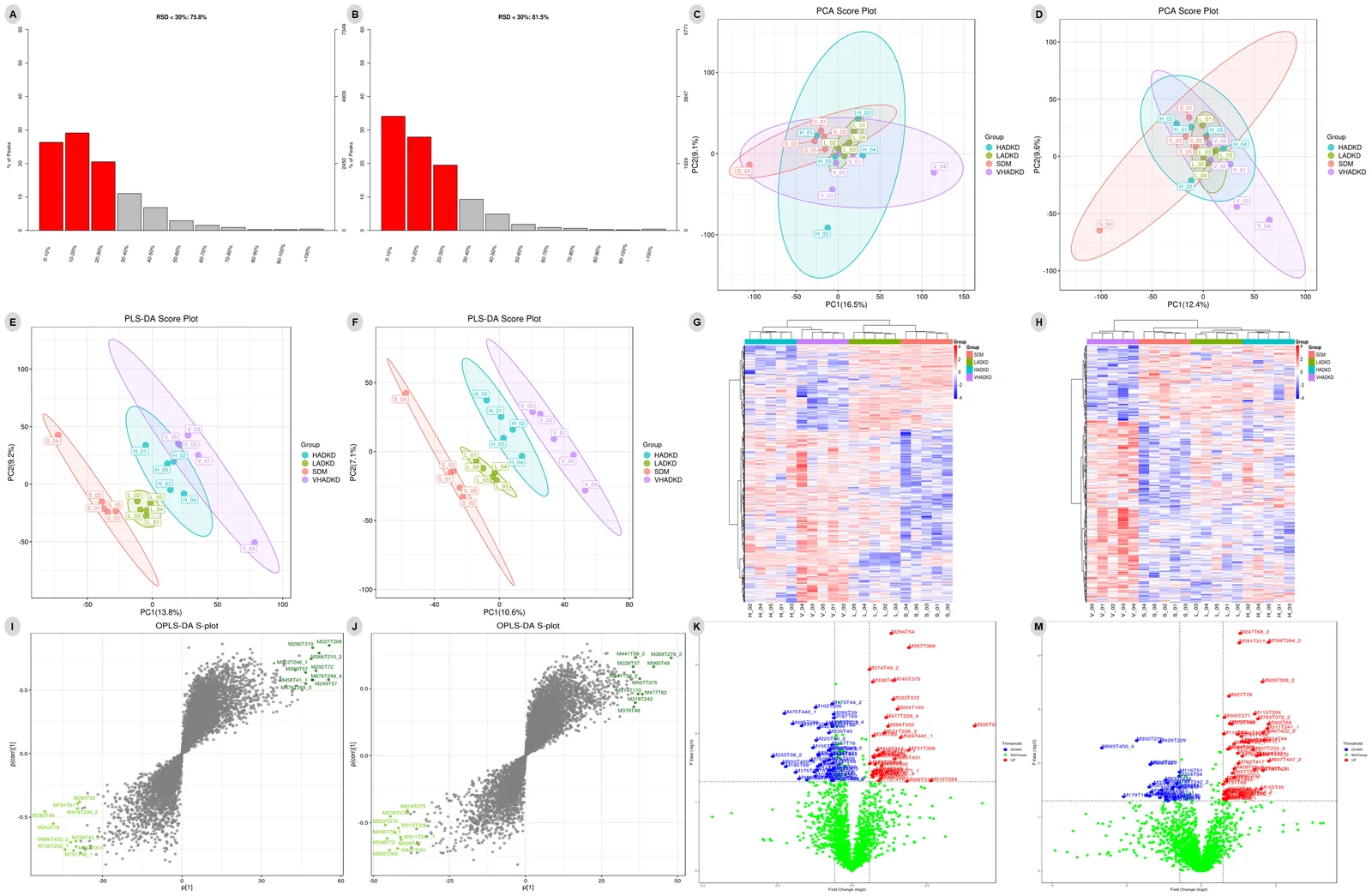
Metabonomic profiling focused on the LADKD cohort. **(A, B)** Coefficient of variation (CV) distribution plot for positive and negative ion primary metabolites; **(C, D)** PCA plot for positive and negative ion primary metabolites; **(E, F)** PLS-DA plot for positive and negative ion primary metabolites; **(G, H)** Hierarchical clustering analysis (HCA) plot for positive and negative ion primary metabolites; **(I, J)** Orthogonal partial least squares discriminant analysis (OPLS-DA) scores plot for positive and negative ion primary metabolites; **(K)** Volcano plot between LADKD and simple DM; **(M)** Volcano plot between LADKD and HADKD/VADKD.

### Integrated proteomic-metabolomic analysis and differential protein screening

Integrated analysis based on 11 databases found that that differential proteins and metabolites in the LADKD group were significantly enriched in 33 metabolic pathways, with five metabolic maps showing the most significant perturbation (**Figure 4A**, **Supplementary Table 1**): Metabolism (Reactome Database), Cysteine biosynthesis (HumanCyc Database), Retinol biosynthesis (HumanCyc Database), Phase II conjugation (Reactome Database), and Selenoamino acid metabolism (SMPDB Database).

**Figure 4:**
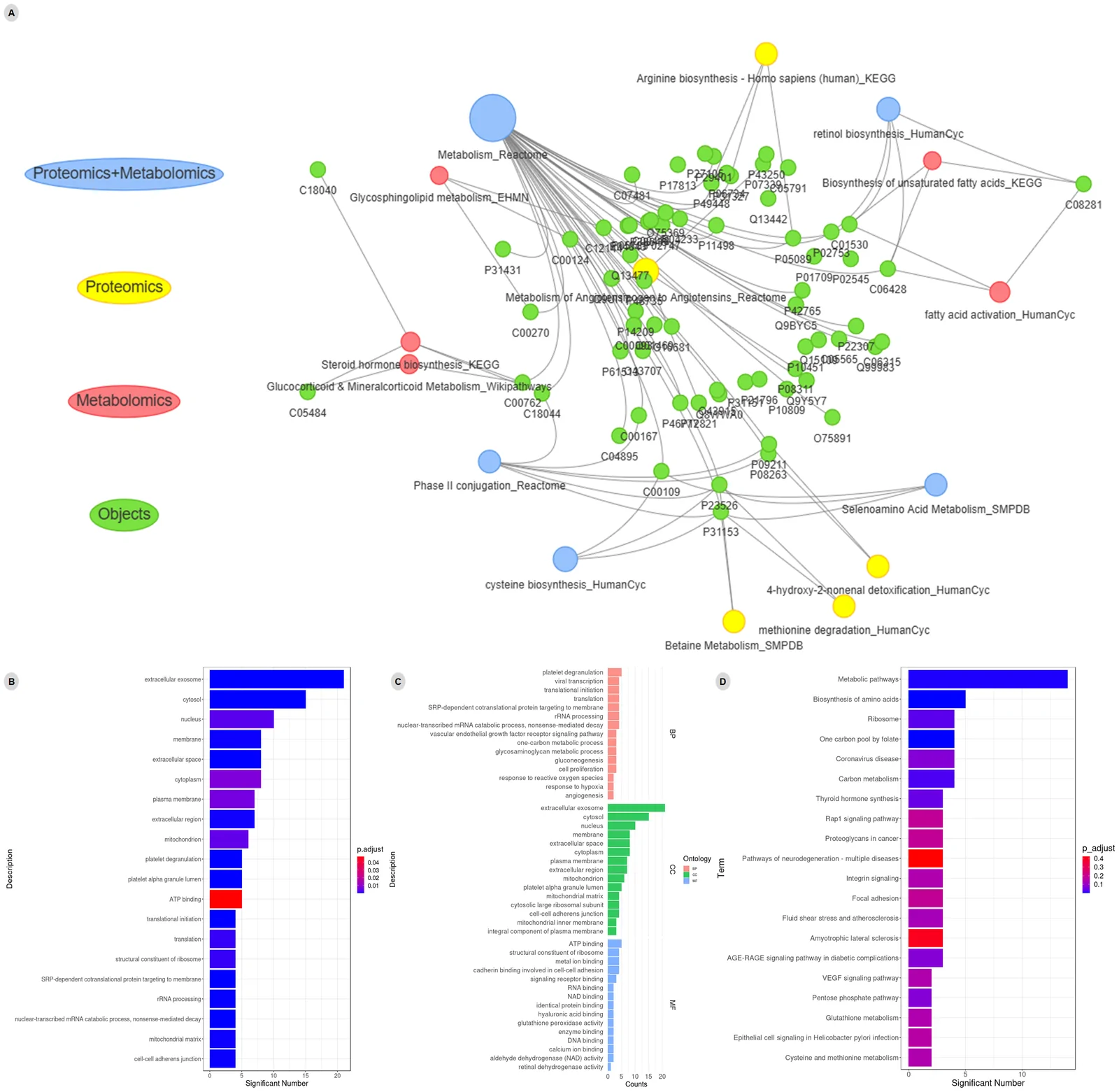
Proteomic-metabolomic integrated network and functional enrichment. **(A)** Integrated proteomic-metabolomic network plot; **(B)** GO functional enrichment; **(C)** GO annotation trinity bar chart; **(D)** KEGG functional enrichment.

The integrated analysis identified 29 differential proteins: 8 upregulated and 21 downregulated. GO enrichment analysis (**Figures 4B, C**) revealed that these proteins are over-represented in extracellular exosome, cytosol, nucleus, membrane, extracellular space, and cytoplasm (adj. p-value<0.05). The over-representation falls into three categories: 1) biological processes: platelet degranulation, viral transcription, translational initiation; 2) cellular components: extracellular exosome, cytosol, nucleus; 3) molecular functions: ATP binding, structural constituent of ribosome, metal ion binding, cadherin binding in cell-cell adhesion (**Supplementary Table 2**). KEGG enrichment analysis (**Figure 4D**) indicated that these proteins are primarily engaged in key processes including Metabolic pathways, Biosynthesis of amino acids, Ribosome, One carbon pool by folate, Coronavirus disease, and Carbon metabolism (**Supplementary Table 3**).

To address multicollinearity, a total of 16 iterative collinearity diagnostic procedures were conducted to eliminated the highest VIF protein per iteration until all VIF fell below 10, ultimately retaining 14 differentially expressed proteins (**Table 2**). Subsequent backward elimination procedure with the threshold of P>0.10 was performed on these 14 differentially expressed proteins, yielding a final panel of 10 candidate proteins with significant clinical relevance: VEGFD, ALDH1A1, PLG, STA1, AHCY, SDC4, RPL37A, RAC1, B3GALT6, and OMD.

**Table 2:** Multicollinearity detection and parameter significance analysis of differentially expressed proteins.

Proteins	First collinearity check					Last collinearity check					Parameter significance test				
	Beta ± SE	Std. Beta	t	P	VIF	Beta ± SE	Std. Beta	t	P	VIF	Beta ± SE	Std. Beta	t	P	VIF
VEGFD	0.156 ± 0.574	0.071	0.272	0.796	3.151	0.805 ± 0.217	0.291	3.703	0.014	3.635	1.007 ± 0.146	0.364	6.911	<0.001	1.928
ALDH1L1	-0.557 ± 0.291	-0.936	-1.915	0.114	11.196	NA	NA	NA	NA	NA	NA	NA	NA	NA	NA
ALDH1A1	-0.008 ± 0.756	-0.004	-0.010	0.992	7.891	0.278 ± 0.132	0.151	2.113	0.088	2.995	0.281 ± 0.091	0.152	3.087	0.013	1.686
PLG	1.551 ± 1.108	0.483	1.399	0.221	5.592	0.268 ± 0.294	0.083	0.910	0.405	4.927	0.638 ± 0.158	0.199	4.028	0.003	1.688
ALB	-1.299 ± 1.718	-0.259	-0.756	0.483	5.506	NA	NA	NA	NA	NA	NA	NA	NA	NA	NA
ALDOA	0.411 ± 2.905	0.237	0.141	0.893	131.429	NA	NA	NA	NA	NA	NA	NA	NA	NA	NA
SERPINE1	0.334 ± 0.527	0.121	0.633	0.554	1.703	NA	NA	NA	NA	NA	NA	NA	NA	NA	NA
GPX1	0.604 ± 1.254	0.323	0.482	0.650	21.033	NA	NA	NA	NA	NA	NA	NA	NA	NA	NA
SLC2A3	-0.744 ± 0.905	-0.598	-0.822	0.449	24.874	NA	NA	NA	NA	NA	NA	NA	NA	NA	NA
PC	0.467 ± 0.632	0.390	0.739	0.493	13.070	NA	NA	NA	NA	NA	NA	NA	NA	NA	NA
CKMT1A	-0.098 ± 1.048	-0.035	-0.093	0.929	6.504	0.167 ± 0.194	0.075	0.861	0.429	4.500	NA	NA	NA	NA	NA
SRC	0.789 ± 1.465	0.473	0.539	0.613	36.091	NA	NA	NA	NA	NA	NA	NA	NA	NA	NA
VCAN	0.350 ± 0.575	0.153	0.608	0.570	2.970	0.133 ± 0.177	0.058	0.756	0.484	3.513	NA	NA	NA	NA	NA
GSTA1	1.046 ± 0.493	0.457	2.121	0.087	2.173	0.623 ± 0.173	0.272	3.598	0.016	3.360	0.658 ± 0.128	0.287	5.156	0.001	2.151
GPD1	0.005 ± 0.144	0.005	0.035	0.974	5.501	-0.009 ± 0.084	-0.009	-0.101	0.923	4.319	NA	NA	NA	NA	NA
GPX3	-0.190 ± 0.292	-0.110	-0.652	0.550	7.263	NA	NA	NA	NA	NA	NA	NA	NA	NA	NA
AHCY	-0.308 ± 0.197	-0.570	-1.565	0.193	33.894	0.773 ± 0.176	0.275	4.397	0.007	2.293	0.664 ± 0.143	0.236	4.630	0.001	1.801
TKT	0.315 ± 0.202	0.450	1.555	0.195	21.476	NA	NA	NA	NA	NA	NA	NA	NA	NA	NA
MAT2A	0.169 ± 0.184	0.246	0.918	0.411	18.342	NA	NA	NA	NA	NA	NA	NA	NA	NA	NA
CPS1	-0.381 ± 0.410	-0.405	-0.929	0.406	48.616	NA	NA	NA	NA	NA	NA	NA	NA	NA	NA
SDC4	-0.004 ± 0.276	-0.002	-0.015	0.989	3.578	0.317 ± 0.197	0.136	1.611	0.168	4.174	0.387 ± 0.137	0.166	2.828	0.020	2.380
RPL15	-0.125 ± 0.369	-0.063	-0.338	0.752	9.007	NA	NA	NA	NA	NA	NA	NA	NA	NA	NA
RPL37A	-0.748 ± 0.306	-0.770	-2.442	0.071	25.419	-0.833 ± 0.135	-0.856	-6.159	0.002	8.356	-0.797 ± 0.092	-0.820	-8.651	<0.001	6.233
RAC1	-0.534 ± 0.663	-0.156	-0.806	0.465	9.539	0.816 ± 0.254	0.238	3.213	0.024	3.214	0.930 ± 0.204	0.271	4.564	0.001	2.444
RPL24	-0.448 ± 0.246	-0.360	-1.819	0.143	10.044	NA	NA	NA	NA	NA	NA	NA	NA	NA	NA
B3GALT6	0.469 ± 0.253	0.238	1.858	0.137	4.200	0.317 ± 0.110	0.299	2.872	0.035	6.379	0.433 ± 0.073	0.409	5.919	<0.001	3.311
RETN	0.377 ± 0.301	0.231	1.251	0.279	8.719	NA	NA	NA	NA	NA	NA	NA	NA	NA	NA
OMD	0.214 ± 0.243	0.179	0.879	0.429	10.603	0.428 ± 0.137	0.358	3.118	0.026	7.759	0.469 ± 0.105	0.392	4.459	0.002	5.365
LYVE1	-0.244 ± 0.270	-0.231	-0.906	0.416	16.652	0.216 ± 0.162	0.110	1.339	0.238	3.950	NA	NA	NA	NA	NA

SE, standard error; Std. Beta, standardized Beta; VIF, variance inflation factor. NA, no data due to exclusion of the independent variable.

### Preliminary validation for candidate protein

Since HADKD and VADKD show significant clustering overlap at the proteomic level, this study combines them into albuminuric DKD to better achieve the goal of distinguishing LADKD. The gender ratio, age distribution, and ELISA results for the 10 candidate differential proteins in the validation cohort are shown in **Table 3**. Within the training set, after screening via the optimal λ.1SE method of LASSO regression (**Figures 5A, B**) and the R^2^, AdjR^2^, BIC, and Cp criteria of Best Subset Selection (**Figure 5C**), 8 feature variables were consistently identified to distinguish simple DM, LADKD, and albuminuric DKD, including age, VEGFD, ALDH1A1, GSTA1, AHCY, RPL37A, B3GALT6, and OMD.

**Table 3:** Demographic and laboratory data of the validation cohort.

Indicator	DM (n=270)	LADKD (n=270)	HADKD/VADKD (n=270)	χ2	P
Male [n (%)]	148(61.7)	120(50.0) ^Δ^	156(65.0) ^▲^	12.300	0.002
Age (years)	54(48 to 61)	69(61 to 77) ^Δ^	60(51 to 69) ^Δ▲^	163.236	<0.001
AHCY (ng/mL)	2.16(1.65 to 4.07)	1.97(1.67 to 2.84) ^Δ^	2.39(1.64 to 4.18) ^▲^	7.643	0.021
ALDH1A1 (pg/mL)	459(384 to 521)	470(418 to 560) ^Δ^	596(420 to 755) ^Δ▲^	68.120	<0.001
B3GALT6 (IU/L)	11.0(7.1 to 14.1)	10.8(8.9 to 12.3)	14.6(11.1 to 18.3) ^Δ▲^	83.145	<0.001
GSTA1 (IU/L)	3.91(2.83 to 5.10)	3.25(2.61 to 3.70) ^Δ^	2.27(1.73 to 3.07) ^Δ▲^	175.478	<0.001
OMD (ng/mL)	1.62(1.08 to 2.62)	2.38(1.20 to 6.80) ^Δ^	2.78(1.39 to 5.85) ^Δ^	31.661	<0.001
PLG (μg/mL)	446(369 to 570)	491(399 to 567) ^Δ^	432(369 to 528) ^▲^	12.102	0.002
RAC1 (mU/L)	88.5(70.7 to 100.2)	89.4(77.7 to 101.4)	96.2(77.7 to 122.5) ^Δ▲^	19.625	<0.001
RPL37A (pg/mL)	310(198 to 418)	185(139 to 269) ^Δ^	180(124 to 273) ^Δ^	103.920	<0.001
SDC4 (ng/mL)	10.4(7.5 to 18.6)	9.8(7.0 to 16.1)	16.0(10.5 to 30.6) ^Δ▲^	55.665	<0.001
VEGFD (pg/mL)	33.2(20.1 to 61.9)	36.8(17.9 to 52.3)	63.5(44.8 to 99.6) ^Δ▲^	94.039	<0.001

^Δ^ LADKD or ADKD vs. DM, Padj<0.05; ^▲^ ADKD vs. LADKD, Padj<0.05.

**Figure 5:**
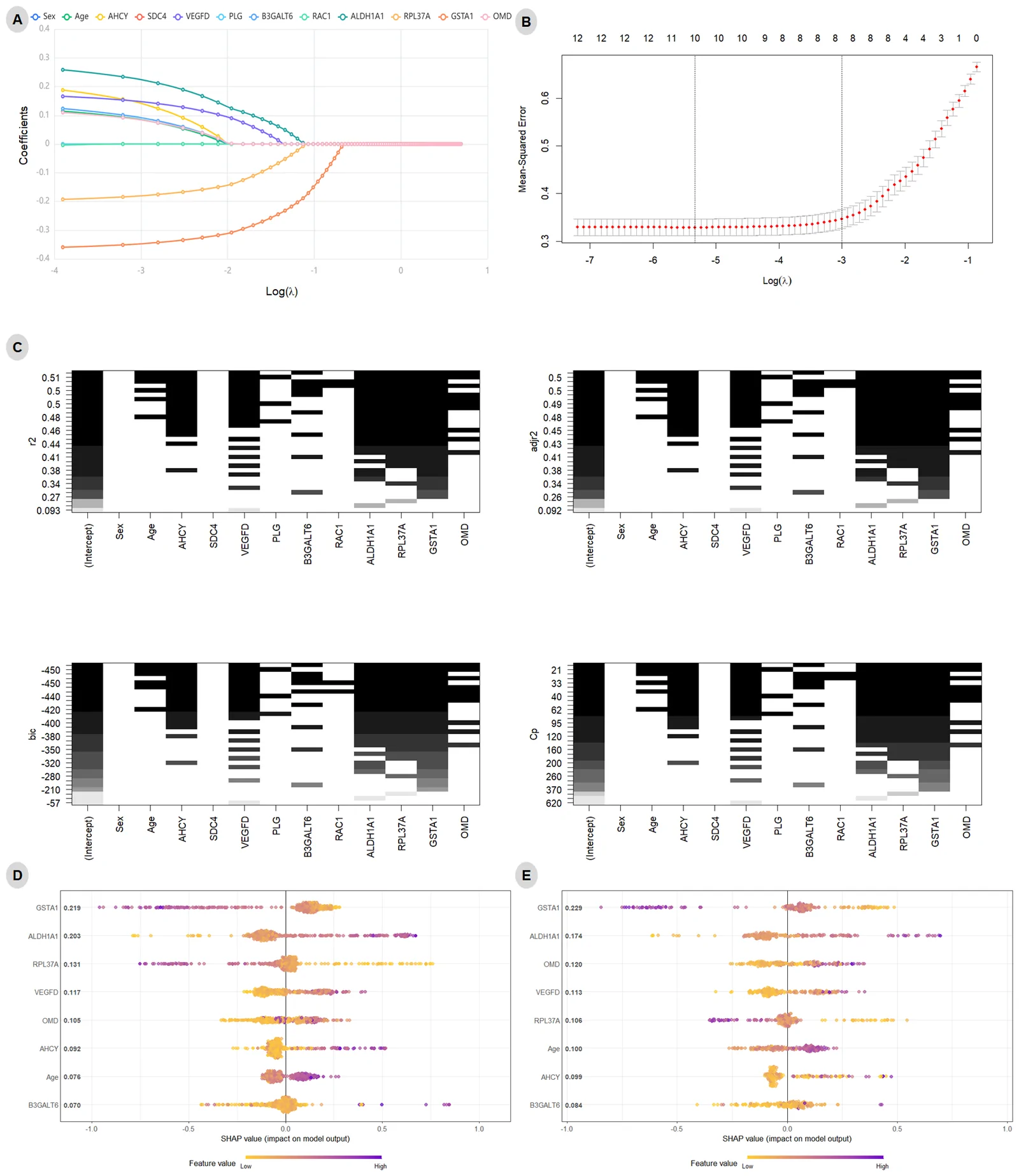
Featured protein screening and enrichment analysis. **(A)** LASSO coefficient path plot; **(B)** LASSO regularization path plot; **(C)** Best subset selection with R2, AdjR2, BIC, and Cp criteria; **(D)** SHAP summary plot in training set; **(E)** SHAP summary plot in testing set. Using sex, age and 10 differentially expressed proteins, LASSO regression screened 8 clinically relevant features consistent with the result of four Best Subset Selection methods. GSTA1 and ALDH1A1 had the highest SHAP values among the 8 features.

**Table 4** presents the diagnostic performance of nine common machine learning classifiers tested on these feature variables. Four models achieved accuracy, recall, precision, F1 score and AUC above 0.9 on both the training and testing sets, proving these features had strong discriminatory diagnostic capability. But only the XGBoost classifier model had all performance metrics above 0.8 on the cross-validation set, indicating its superior generalization ability. The SHAP-based feature importance rankings of the training set were GSTA1> ALDH1A1> RPL37A> VEGFD> OMD> AHCY> Age> B3GALT6 (corresponding SHAP ± SE values 0.219 ± 0.033, 0.203 ± 0.037, 0.131 ± 0.046, 0.117 ± 0.025, 0.105 ± 0.027, 0.092 ± 0.025, 0.076 ± 0.026, 0.07 ± 0.016; **Figure 5D**); Those in the test set were GSTA1> ALDH1A1> OMD> VEGFD> RPL37A> Age > AHCY> B3GALT6 (corresponding SHAP ± SE values 0.229 ± 0.050, 0.174 ± 0.038, 0.120 ± 0.023, 0.113 ± 0.026, 0.106 ± 0.024, 0.100 ± 0.021, 0.099 ± 0.016, 0.084 ± 0.026; **Figure 5E**). Notably, GSTA1 and ALDH1A1 consistently ranked first and second highest SHAP values across both datasets, and their feature importance ranked within the top four across the four classifiers with higher AUC values (**Figure 6**), indicating their robust and dominant contribution to model prediction.

**Table 4:** Performance evaluation of nine common machine learning classifier models.

ML algorithms	Accuracy	Recall	Precision	F1 Score	AUC (95%CI)
XGBoost classifier					
Training set	1.000	1.000	1.000	1.000	1.000(1.000 to 1.000)
Cross-validation set	0.889	0.889	0.889	0.889	0.843(0.816 to 0.870)
Testing Set	0.908	0.908	0.910	0.908	0.982(0.965 to 0.992)
GBDT classifier					
Training set	1.000	1.000	1.000	1.000	1.000(1.000 to 1.000)
Cross-validation set	0.841	0.841	0.841	0.841	0.690(0.651 to 0.729)
Testing Set	0.901	0.901	0.902	0.901	0.982(0.965 to 0.990)
RF classifier					
Training set	0.992	0.992	0.992	0.992	1.000(1.000 to 1.000)
Cross-validation set	0.881	0.881	0.882	0.881	0.741(0.706 to 0.776)
Testing Set	0.912	0.912	0.914	0.913	0.984(0.954 to 0.999)
Extra trees classifier					
Training set	0.972	0.972	0.973	0.972	0.998(0.992 to 1.000)
Cross-validation set	0.867	0.867	0.871	0.867	0.727(0.675 to 0.779)
Testing Set	0.917	0.917	0.924	0.918	0.980(0.940 to 1.000)
AdaBoost classifier					
Training set	0.847	0.847	0.847	0.847	0.911(0.870 to 0.936)
Cross-validation set	0.769	0.769	0.771	0.770	0.637(0.601 to 0.673)
Testing Set	0.779	0.779	0.779	0.779	0.875(0.799 to 0.944)
KNN classifier					
Training set	0.809	0.809	0.812	0.809	0.946(0.893 to 0.959)
Cross-validation set	0.696	0.696	0.704	0.696	0.583(0.526 to 0.640)
Testing Set	0.645	0.645	0.662	0.646	0.838(0.734 to 0.867)
Logistic regression					
Training set	0.761	0.761	0.763	0.762	0.902(0.858 to 0.929)
Cross-validation set	0.728	0.728	0.728	0.728	0.651(0.608 to 0.694)
Testing Set	0.710	0.710	0.715	0.705	0.868(0.806 to 0.920)
Naive bayes classifier					
Training set	0.694	0.694	0.695	0.694	0.858(0.811 to 0.893)
Cross-validation set	0.686	0.686	0.687	0.686	0.535(0.491 to 0.579)
Testing Set	0.710	0.710	0.733	0.713	0.869(0.790 to 0.919)
SVM classifier					
Training set	0.421	0.421	0.584	0.423	0.523(0.507 to 0.529)
Cross-validation set	0.406	0.406	0.409	0.392	0.538(0.490 to 0.586)
Testing Set	0.438	0.438	0.555	0.430	0.479(0.458 to 0.512)

ML, machine learning; XGBoost, extreme gradient boosting; GBDT, gradient boosted decision tree; RF, random forest; AdaBoost, adaptive boosting; KNN, K-nearest neighbor; SVM, support vector machine.

**Figure 6:**
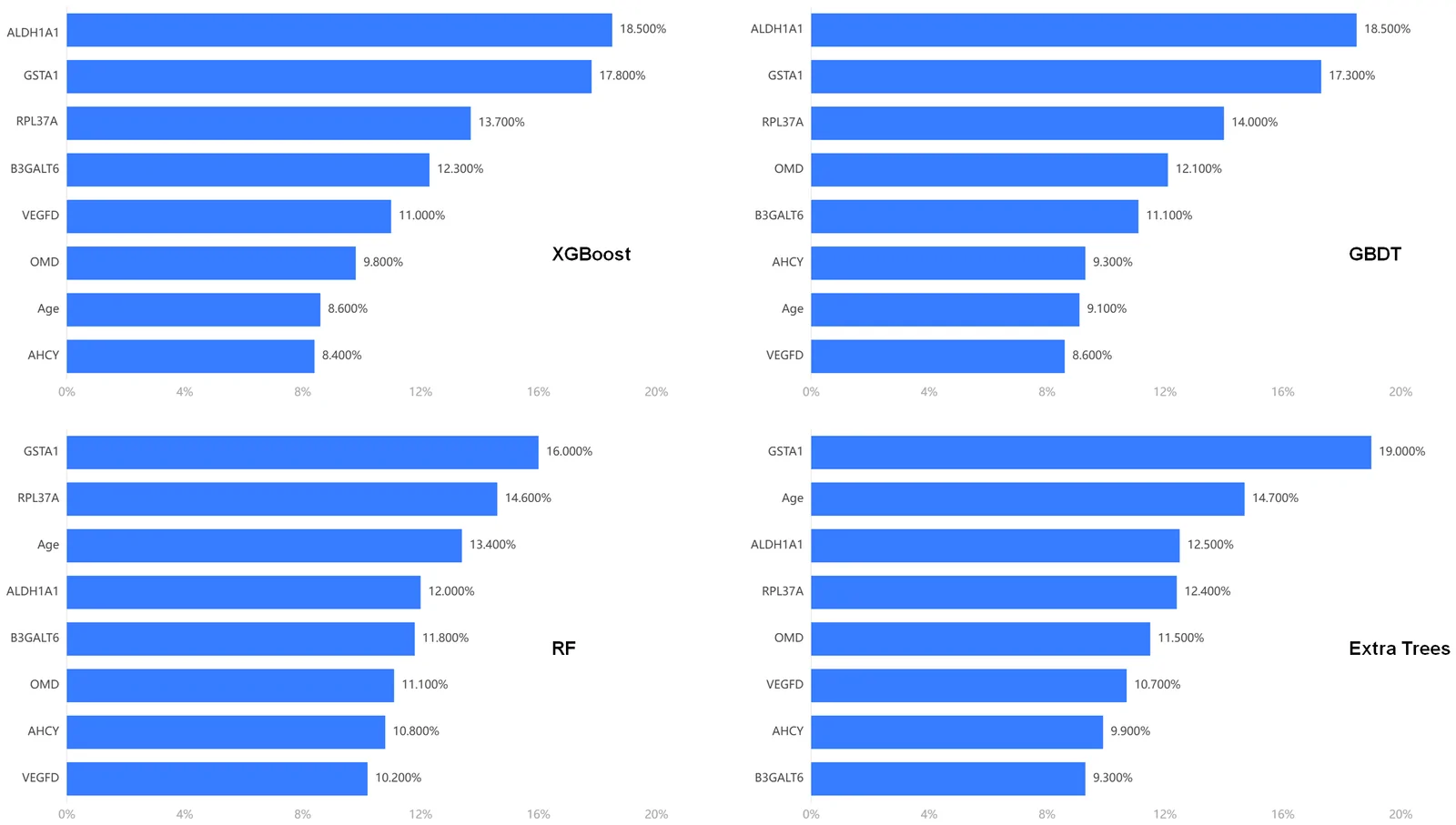
Feature importance ranking across the four classifiers with higher AUC values. XGBoost, extreme gradient boosting; GBDT, gradient boosted decision tree; RF, random forest. GSTA1 and ALDH1A1 feature importances ranked within the top four across the four classifiers, indicating their robust and dominant contribution to model prediction.

## Discussion

The concept of low albuminuria has been proposed for nearly two decades, yet diagnosing LADKD remains challenging, likely due to the current lack of specific diagnostic biomarkers. This study provides the following key findings that update our understanding of non-albuminuric DKD: 1) The subdivided NADKD is extremely rare among non-albuminuric DKD subtypes, whereas LADKD accounts for the majority, suggesting a possible potential trajectory from DM to LADKD to albuminuric DKD. Notably, both DKD groups were of advanced age, which supports the possibility of multi-pathway disease potential trajectory. 2) Integrated proteomic-metabolomic analysis revealed a metabolic-signal-structural multidimensional transition in LADKD patients, covering widespread metabolic pathway disturbances, aberrant signal transduction activation, protein complex remodeling, and dysregulated molecular control networks. These changes collectively drive disease progression from glucose metabolism abnormalities to diabetic nephropathy, accompanied by renal structural and functional damage. 3) Preliminary validation revealed the diagnostic potential of multiple differentially expressed proteins for distinguishing LADKD from DM and albuminuric DKD, particularly GSTA1 and ALDH1A1.

Numerous epidemiological studies have reported that non-albuminuric DKD accounts for 20% to 70% of all DKD cases (10, 32). However, large-scale standardized epidemiological investigations that further stratify non-albuminuric DKD into UACR<10mg/g (subdivided NADKD) and UACR 10-29mg/g (LADKD) subgroups remain scarce, particularly with a scarcity of nationwide data based on this classification. In our first-phase cross-sectional survey, the prevalence of subdivided NADKD was extremely low, accounting for only 0.7% of total diabetic population and 1.6% of all DKD cases. In contrast, LADKD constituted the overwhelming majority of non-albuminuric DKD cases, with a prevalence of 6.7% among diabetic patients and 14.0% among all DKD patients. The prevalence of LADKD is consistent with some studies reporting 10%-20% (33, 34). Overall, consistent with the prior NADKD population classification, the subdivided NADKD+LADKD cohort in our study includes 7.4% DM and 15.6% DKD. This distribution may reflect selection bias from the single-center design and exclusive endocrinology department recruitment. During the three-month omics sample collection from newly diagnosed patients, one subdivided NADKD case and 28 LADKD cases were collected, indirectly confirming the above prevalence distribution. As subdivided NADKD diagnosis cannot rule out misclassification from methodological flaws in UACR measurement (35) and bias in eGFR estimation (36), larger multicenter validation is recommended. Meanwhile, low-grade albuminuria is confirmed as an independent risk factor for the progression of diabetic kidney disease and cardiovascular events (37–39). Therefore, future non-albuminuric DKD studies should prioritize LADKD, for its better accessibility and higher clinical relevance.

Non-invasive, proteinuria-independent biomarkers are urgently needed to improve DKD management. Using integrated proteomic-metabolomic analysis, this study identified 29 differential proteins in LADKD patients, enriched across five metabolic maps involving metabolic dysregulation, aberrant signal transduction, protein complex remodeling, and disrupted molecular control networks. Further screening narrowed them to seven core proteins (VEGFD, ALDH1A1, GSTA1, AHCY, RPL37A, B3GALT6, and OMD), which demonstrated strong diagnostic performance for LADKD across multiple machine learning classifiers. Among them, the XGBoost model showed the best generalization ability and consistently ranked GSTA1 and ALDH1A1 as the top two features by importance.

GSTA1 is a key member of the glutathione S-transferase alpha class family. It catalyzes the conjugation of glutathione with electrophilic compounds and plays a role in antioxidant defense and metabolic regulation (40). In humans, GSTA1 is primarily expressed in proximal tubular epithelial cells and hepatocytes (41), and is released into blood and urine upon kidney injury. Existing studies on diabetic patients mainly focus on glutathione S-transferase and other subtypes (40, 42), with little attention given to GSTA1. Even when included, results are negative (43). However, multiple studies demonstrate that GSTA1 holds diagnostic value for early tubular injury, particularly in acute kidney injury and drug-induced kidney injury (44, 45). Therefore, it can be hypothesized that abnormal expression of GSTA1 in DM patients may impair local renal antioxidant defenses, thereby exacerbating oxidative stress-mediated kidney damage. Our findings indicate that serum GSTA1 levels progressively increase with worsening UACR in diabetic patients, and that GSTA1 is the most promising diagnostic biomarker among all differentially expressed proteins identified in LADKD, suggesting its potential as a supplement for early DKD screening.

ALDH1A1, a core member of the aldehyde dehydrogenase superfamily, catalyzes aldehyde oxidation into carboxylic acids, mediating retinoic acid synthesis, aldehyde detoxification, and antioxidant stress response (46, 47). Studies indicate it has unique protective effects in renal tubular repair and oxidative stress mitigation: upregulation in proximal tubule cells promotes retinoic acid synthesis, activates the RAR/RXR pathway, and drives tubular epithelial cell regeneration and repair (48); it also converts toxic aldehydes accumulated via lipid peroxidation into non-toxic byproducts to maintain mitochondrial function and cell membrane integrity (49). In diabetic patients, high-glucose-induced oxidative stress and oxidative modification reduce ALDH1A1 activity, aggravating aldehyde accumulation, promoting advanced glycation end product (AGE) formation, thus accelerating glomerular and tubular damage (50). To our knowledge, most evidence in kidney disease comes from basic research and animal models, with no large cohort studies to validate its diagnostic and prognostic value for LADKD. Further research is needed before it can be translated into a clinical biomarker.

Some studies have reported that the other five differential proteins also are linked to kidney injury (VEGFD (51), AHCY (52), RPL37A (53), B3GALT6 (54), and OMD (55)), but few focus on the diabetic population. In this study, VEGFD and B3GALT6 were only significantly elevated in the albuminuric DKD group, so their clinical use is likely restricted to distinguish albuminuric DKD, with limited ability to LADKD. RPL37A was higher and OMD was lower in both LADKD and albuminuric DKD groups, with no significant intergroup difference. Thus, they can only distinguish the existence of DKD, not separate the two subtypes. AHCY levels was lower in the LADKD group than in the simple DM and albuminuric DKD groups, contributing to LADKD diagnosis. However, AHCY had lower feature importance in the XGBoost classifier. Overall, these five proteins have relatively limited potential as LADKD biomarkers, while GSTA1 and ALDH1A1 show better discriminatory capacity and clinical relevance for early LADKD identification.

Strengths and limitations: Main strengths are using mGFR to validate subdivided NADKD and LADKD classification, and conducting integrated proteomic-metabolomic analysis on 11 metabolic pathway databases. Limitations include: rare subdivided NADKD cases were excluded from omics analysis; potential bias from semi-quantitative mass spectrometry; limited statistical power from small sample omics analysis; and uncertain generalizability from single-center clinical validation. Additionally, the links between GSTA1/ALDH1A1 and tubular injury along with between LADKD and DKD progression are still hypothetical and require validation through rigorous biomarker validation, mechanistic studies, and longitudinal studies. Future research can explore their pathogenic mechanisms via animal experiments and evaluate diagnostic potential through cohort studies. In addition, subdivided NADKD also requires dedicated research.

## Conclusion

Diagnosis of non-albuminuric DKD and LADKD remains challenging due to the absence of widely accepted, sensitive and specific biomarkers. This study integrated proteomic-metabolomic analysis with statistical screening to identify age and seven potential differentially expressed proteins for distinguishing LADKD, which showed excellent diagnostic performance across multiple machine learning classifiers. These proteins may contribute separately to detect DKD existence (RPL37A and OMD), diagnose albuminuric DKD (VEGFD and B3GALT6) or LADKD (AHCY), and differentiate simple DM, LADKD and albuminuric DKD (GSTA1 and ALDH1A1). GSTA1 and ALDH1A1 contributed the most to this differential diagnosis. This study provides a promising detection method for managing chronic kidney injury progression in diabetic patients, enriches potential therapeutic strategies for DKD management, and notes that further mechanistic research and diagnostic validation are warranted.

## Data Availability

The datasets presented in this study can be found in online repositories. The names of the repository/repositories and accession number(s) can be found below: http://doi.org/10.5281/zenodo.21830722, 21830722.
