# Supplementary material for: Integrated omics analysis identifies GSTA1 and ALDH1A1 as candidate biomarkers for low-level albuminuric diabetic kidney disease: a mixed prospective-retrospective cross-sectional study
Source: Front Endocrinol (Lausanne). 2026 Oct 19;17:1978947. doi: 10.3389/fendo.2026.1978947 (PMC13724592; doi:10.3389/fendo.2026.1978947)
Supplement: Supplementary file 1 [file Table1.docx]

**Supplementary Table 1. Significantly Altered Metabolic Pathways in Integrated Proteomic-metabolomic Network (Adjusted Joint-P <0.05).**

| Pathway Name | Path source | Proteins | | | | Metabolites | | | | Joint Padjust |
| --- | --- | --- | --- | --- | --- | --- | --- | --- | --- | --- |
|  |  | Component Ratio | Background Ratio | Padjust | Matched proteins | Component  Ratio | Background  Ratio | Pdjust | Matched  metabolites |  |
| Methionine De Novo and Salvage Pathway | Wikipathways | 2/147 | 32/21644 | 0.119876 | P23526/P31153 | 4/40 | 41/4289 | 0.181644 | C15606/C00315/  C00018/C00147 | 0.000269 |
| Folate Metabolism | Wikipathways | 6/147 | 129/21644 | 0.00407 | P00747/P02768/  P05121/P07203/  P22352/P23526 | 2/40 | 67/4289 | 0.356887 | C00018/C03838 | 0.000763 |
| Cysteine biosynthesis | HumanCyc | 2/147 | 15/21644 | 0.043042 | P23526/P31153 | 2/40 | 17/4289 | 0.290943 | C00155/C00109 | 0.001094 |
| Cystathionine Beta-Synthase Deficiency | SMPDB | 2/147 | 34/21644 | 0.127479 | P23526/P31153 | 3/40 | 42/4289 | 0.195724 | C00315/C00018/  C00109 | 0.002719 |
| Glycine N-methyltransferase Deficiency | SMPDB | 2/147 | 34/21644 | 0.127479 | P23526/P31153 | 3/40 | 42/4289 | 0.195724 | C00315/C00018/  C00109 | 0.002719 |
| Homocystinuria-megaloblastic anemia due to defect in cobalamin metabolism, cblG complementation type | SMPDB | 2/147 | 34/21644 | 0.127479 | P23526/P31153 | 3/40 | 42/4289 | 0.195724 | C00315/C00018/  C00109 | 0.002719 |
| Hypermethioninemia | SMPDB | 2/147 | 34/21644 | 0.127479 | P23526/P31153 | 3/40 | 42/4289 | 0.195724 | C00315/C00018/  C00109 | 0.002719 |
| Methionine Adenosyltransferase Deficiency | SMPDB | 2/147 | 34/21644 | 0.127479 | P23526/P31153 | 3/40 | 42/4289 | 0.195724 | C00315/C00018/  C00109 | 0.002719 |
| Methionine Metabolism | SMPDB | 2/147 | 34/21644 | 0.127479 | P23526/P31153 | 3/40 | 42/4289 | 0.195724 | C00315/C00018/  C00109 | 0.002719 |
| Methylenetetrahydrofolate Reductase Deficiency (MTHFRD) | SMPDB | 2/147 | 34/21644 | 0.127479 | P23526/P31153 | 3/40 | 42/4289 | 0.195724 | C00315/C00018/  C00109 | 0.002719 |
| S-Adenosylhomocysteine (SAH) Hydrolase Deficiency | SMPDB | 2/147 | 34/21644 | 0.127479 | P23526/P31153 | 3/40 | 42/4289 | 0.195724 | C00315/C00018/  C00109 | 0.002719 |
| methionine salvage cycle III | HumanCyc | 1/147 | 17/21644 | 0.309958 | P31153 | 3/40 | 25/4289 | 0.181644 | C15606/C00315/  C00147 | 0.002926 |
| methionine degradation | HumanCyc | 2/147 | 10/21644 | 0.022914 | P23526/P31153 | 1/40 | 12/4289 | 0.350948 | C00155 | 0.003706 |
| Selenoamino Acid Metabolism | SMPDB | 2/147 | 22/21644 | 0.072476 | P23526/P31153 | 2/40 | 28/4289 | 0.319886 | C00018/C00109 | 0.00452 |
| Selenium Micronutrient Network | Wikipathways | 5/147 | 138/21644 | 0.027223 | P00747/P02768/  P05121/P07203/  P22352 | 2/40 | 106/4289 | 0.416453 | C00018/C06315 | 0.00988 |
| Alpha9 beta1 integrin signaling events | PID | 3/147 | 57/21644 | 0.059024 | O43915/P12931/  P63000 | 1/40 | 13/4289 | 0.350948 | C00315 | 0.011629 |
| Methylenetetrahydrofolate Reductase Deficiency (MTHFRD) | SMPDB | 1/147 | 22/21644 | 0.350485 | O75891 | 3/40 | 42/4289 | 0.195724 | C00315/C00018/  C00109 | 0.013215 |
| Spermidine and Spermine Biosynthesis | SMPDB | 1/147 | 13/21644 | 0.274733 | P31153 | 2/40 | 18/4289 | 0.292854 | C00315/C00018 | 0.013802 |
| superpathway of methionine degradation | HumanCyc | 2/147 | 37/21644 | 0.143045 | P23526/P31153 | 2/40 | 36/4289 | 0.319886 | C00155/C00109 | 0.015401 |
| Metabolism of carbohydrates | Reactome | 9/147 | 505/21644 | 0.063455 | P00352/P04075/  P11169/P11498/  P13611/P29401/  P31431/Q96L58/  Q9Y5Y7 | 3/40 | 144/4289 | 0.365506 | C00111/C00018/  C00124 | 0.015411 |
| Selenoamino acid metabolism | Reactome | 5/147 | 199/21644 | 0.082511 | P18621/P23526/  P61313/P61513/  P83731 | 2/40 | 61/4289 | 0.350948 | C00018/C00109 | 0.016823 |
| Metabolism of polyamines | Reactome | 2/147 | 71/21644 | 0.274733 | P12532/P31327 | 3/40 | 60/4289 | 0.319886 | C00315/C00018/  C00147 | 0.019473 |
| glycerol-3-phosphate shuttle | HumanCyc | 1/147 | 4/21644 | 0.144702 | P21695 | 1/40 | 7/4289 | 0.339946 | C00111 | 0.021181 |
| Methionine Cysteine metabolism | INOH | 2/147 | 48/21644 | 0.16943 | P23526/P31153 | 2/40 | 45/4289 | 0.340876 | C00155/C00109 | 0.030127 |
| Selenocysteine synthesis | Reactome | 4/147 | 150/21644 | 0.117196 | P18621/P61313/  P61513/P83731 | 1/40 | 18/4289 | 0.365506 | C00018 | 0.031255 |
| Gluconeogenesis | Reactome | 2/147 | 71/21644 | 0.274733 | P04075/P11498 | 2/40 | 34/4289 | 0.319886 | C00111/C00018 | 0.033913 |
| Glucose-6-phosphate dehydrogenase deficiency | SMPDB | 2/147 | 27/21644 | 0.094012 | P04075/P29401 | 1/40 | 29/4289 | 0.416453 | C00111 | 0.034008 |
| Pentose Phosphate Pathway | SMPDB | 2/147 | 27/21644 | 0.094012 | P04075/P29401 | 1/40 | 29/4289 | 0.416453 | C00111 | 0.034008 |
| Ribose-5-phosphate isomerase deficiency | SMPDB | 2/147 | 27/21644 | 0.094012 | P04075/P29401 | 1/40 | 29/4289 | 0.416453 | C00111 | 0.034008 |
| sarcosine oncometabolite pathway | SMPDB | 2/147 | 28/21644 | 0.099131 | P31153/Q9UI17 | 1/40 | 26/4289 | 0.416453 | C00018 | 0.034008 |
| Transaldolase deficiency | SMPDB | 2/147 | 27/21644 | 0.094012 | P04075/P29401 | 1/40 | 29/4289 | 0.416453 | C00111 | 0.034008 |
| Glycerol Phosphate Shuttle | SMPDB | 1/147 | 6/21644 | 0.16943 | P21695 | 1/40 | 10/4289 | 0.350948 | C00111 | 0.035347 |
| visceral fat deposits and the metabolic syndrome | BioCarta | 1/147 | 22/21644 | 0.350485 | Q9HD89 | 1/40 | 3/4289 | 0.319886 | C00762 | 0.037653 |
| Glycolysis and Gluconeogenesis | Wikipathways | 3/147 | 98/21644 | 0.150889 | P04075/P11169/  P11498 | 1/40 | 16/4289 | 0.362131 | C00111 | 0.03893 |
| Metabolism of ingested SeMet, Sec, MeSec into H2Se | Reactome | 1/147 | 19/21644 | 0.331455 | P23526 | 2/40 | 32/4289 | 0.319886 | C00018/C00109 | 0.040517 |
| Glycolysis Gluconeogenesis | INOH | 2/147 | 86/21644 | 0.321396 | P04075/P11498 | 2/40 | 34/4289 | 0.319886 | C00111/C00124 | 0.042372 |
| Methylenetetrahydrofolate Reductase Deficiency (MTHFRD) | SMPDB | 2/147 | 34/21644 | 0.127479 | P23526/P31153 | 1/40 | 28/4289 | 0.416453 | C00018 | 0.04671 |

Note: A total of 37 metabolic pathways with 4 duplicates is listed in the integrated proteomic-metabolomic network, which significantly altered. These metabolic pathways include 29 differentially expressed proteins.
